# A Study on the Improvement of Walking Characteristics of the Elderly with Vibration Stimuli Applied to the Tibialis Anterior Tendon

**DOI:** 10.1155/2017/5342485

**Published:** 2017-11-26

**Authors:** Kiyoung Kwak, Huigyun Kim, Dongwook Kim

**Affiliations:** ^1^Department of Healthcare Engineering, Graduate School, Chonbuk National University, Jeonju, Republic of Korea; ^2^Department of Biomedical Engineering, College of Engineering, Chonbuk National University, Jeonju, Republic of Korea; ^3^Research Center of Healthcare and Welfare Instrument for Aged, Chonbuk National University, Jeonju, Republic of Korea

## Abstract

The purpose of this study was to identify the gait pattern of the elderly with aging and to analyze the elderly's gait changes by the focal tendon vibratory stimulation. A total of 10 elderly males and 15 young adult males participated in this study. Using 3D motion analysis, we analyzed that difference between the elderly gait and young adults gait and the changes of the elderly gait by applying focal vibratory stimuli. As a result, specifically in the early stance, the elderly's gait was more flexed and the lower extremity extensors of the elderly worked harder. When the focal vibratory stimuli were applied, joint angle of the elderly was induced to that of the young adults. There was a reduction in demands for supporting bodies and progressing gait in the stance phase. This means that focal vibratory stimuli affect the gait of the elderly. Also, the changes of the gait of the elderly varied according to the characteristics of the focal vibratory stimuli. This implies that the activity of the motor may be dependent on vibratory stimuli characteristics.

## 1. Introduction

Walking is one of the most important activities in daily life. Walking is a learned activity in which the moving body is supported successively by one leg and the other [1]. Walking is conducted almost unconsciously, but biomechanical subtasks, such as body support, forward propulsion, and maintaining postural stability, must be successfully performed [2]. Successful biomechanical subtasks require complex and well-coordinated activity of lower extremities muscles. However, for elderly experiencing neurological and physiological changes due to aging, the activity of these lower extremities muscles will be diminished. Therefore, maintaining walking abilities is very important for the elderly.

For this reason, a lot of studies have been conducted on the gait of the elderly to date. The gait of the elderly has the following characteristics: a decreased walking velocity [3], a shorter stride length [4], a reduced force at push-off [5], a flatter foot landing pattern at heel-strike [4], and a decreased range of the motion of the lower limbs [6]. Using the results of the studies that characterize the gait of the elderly, applied research on assistance, improvement, and rehabilitation of the elderly gait is needed.

There is a focal muscle tendon vibratory stimulation that can affect neurological and physiological changes in the elderly. There are a number of studies that have shown that focal muscle tendon vibratory stimulation stimulates somatosensory receptors, resulting in responses to the muscular system [7–9] and the central nervous system [10, 11].

As such, there have been a number of studies using vibration giving helpful results. However, they have some limitations about vibration characteristics, and it is much harder to find studies that are closely related to the elderly. In specific, they do not consider the characteristics of vibration (frequency, intensity) and the individual differences against the vibration. Another limitation is that the biomechanical analyses were not applied to the elderly gait.

The purpose of this study was to identify the gait pattern of the elderly with aging and to analyze the elderly's gait changes by the focal muscle tendon vibratory stimulation.

## 2. Methods

### 2.1. Subjects

15 young adult males (age: 26.4 ± 1.5 year, height: 171.6 ± 2.7 cm, weight: 66.1 ± 5.7 kg) and 10 elderly (age: 70.8 ± 2.8 year, height: 164.1 ± 6.7 cm, weight: 66.1 ± 8.0 kg) participated in this experiment. All subjects had no diseases in their nervous and musculoskeletal system and were capable of gait independently without any assisting devices. This study was approved by Chonbuk National University Institutional Review Board (IRB File No. JBNU 2015-06-012).

### 2.2. Equipment

A small scale linear actuator (0934, Samsung Electro-Mechanics, Korea) was used to apply vibration to the tibialis anterior tendon. In addition, a function generator was used to adjust the frequency and intensity of vibration. To capture the gait, a total of 15 active infrared emitting diode markers were attached to each major joint according to Halen-Hays marker set. To collect the infrared light, a total of 3 position sensors (Optotrak Certus, Northern Digital Inc, Canada) were used. To measure the ground reaction force, a total of 4 force platforms (Bertec Co., Ltd, USA) were used.

### 2.3. Vibratory Stimuli Application

To investigate the changes of the elderly gait in the lower extremity according to the characteristics of the applied vibratory stimuli, the frequency and intensity of the vibration were adjusted and vibration perception threshold was measured on the vibration frequency in the tibialis anterior tendon. Based on measurement result, vibration is applied to the tibialis anterior tendon during gait.

By combining the stimulus site, vibration frequency, and perception threshold, vibratory stimulus condition is set. No vibration is applied, and nonstimulation appeared. Vibration at perception threshold intensity (threshold vibration) and 180 Hz of frequency are applied to tibialis anterior tendon, and TAT_180 Hz_Theshold or T180 Hz_Treshold appears. Vibration at 80% of threshold (subthreshold) and 180 Hz of frequency are applied to tibialis anterior tendon, and TAT_180 Hz_Sub Threshold or T180 Hz_Sub Threshold appears.

### 2.4. Protocol

The subjects walked on flat ground at least 10 m at preferred speed. The focal vibratory stimuli were randomly applied. All the subjects walked 3 times per each stimulus condition.

### 2.5. Analysis

To investigate the profiles of the gait in the lower extremity, a 3D human musculoskeletal system modeling and analysis software (SIMM, MusculoGraphics Inc., USA) was used. The stance phase was set as the period for analysis. The joint angle, joint moment, joint power, and support moment of a lower extremity during a stance phase were chosen as the analysis parameters.

The time history profiles of the angle, moment, power, and support moment are illustrated as the result. Then, our analysis concluded that the profiles of the elderly gait differ from young adults and we analyzed the change in the elderly gait with applied focal vibratory stimuli characteristics.

To analyze improvement effect about joint angle by focal vibratory stimuli, we analyzed the following: (1) mean of the difference between the elderly with nonstimulation and young adults, (2) mean of the difference between the elderly with 180 Hz vibration and young adults, (3) mean of the difference between the elderly with 190 Hz vibration and young adults, and (4) mean of the difference between the elderly with 200 Hz vibration and young adults. As for support moments and joint power, we analyzed the means of time averages of support moments and power during double limb stance phase and single limb stance phase [12]. We also performed paired* T*-test (*p* < 0.05) to examine statistical significance using SPSS 20 (IBM Corp, USA).

## 3. Result

### 3.1. Level Gait in the Elderly and Young Adults

#### 3.1.1. Ankle Joint Profiles

**Figure 1:**
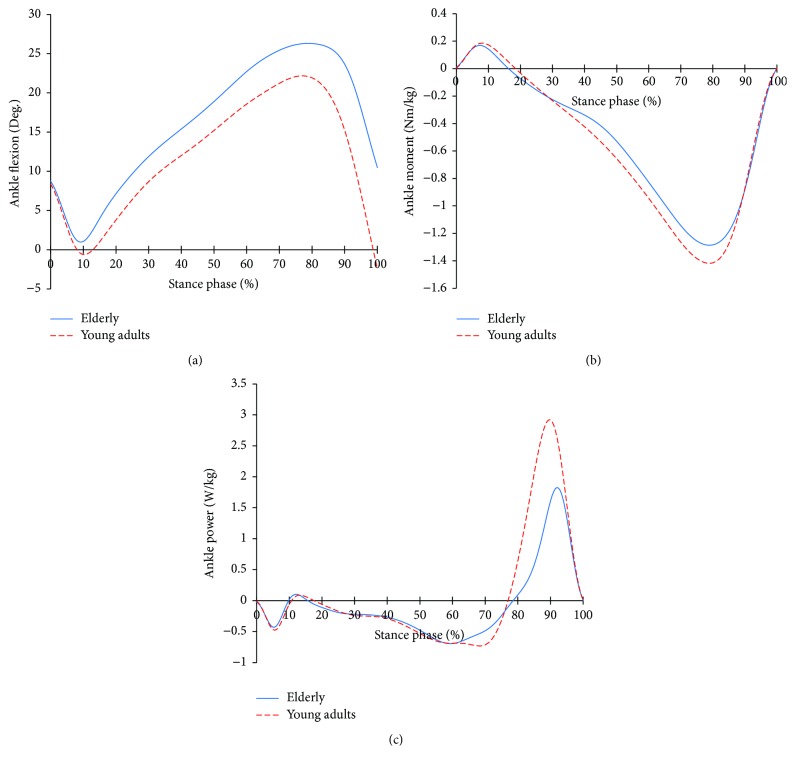
Ankle joint profiles in the elderly and young adults. (a) Ankle joint angle; (b) ankle joint moment; (c) ankle joint power.

Figure 1 shows the ankle joint profiles during the level gait of the two groups. In the flexion angle, the young adults showed plantar flexion and dorsiflexion, while the elderly only showed the dorsiflexion, of which the angle was much greater than that of the young adults. At that moment, both groups showed the same phase. But, the elderly showed the dorsiflexor moment and plantar flexor moment, which were smaller than those of the young adults. In the power, at the loading response phase (LR), phase change timing and magnitude were slightly different from those of the young adults. Also, after 60% of the stance phase, the elderly showed that the phase change was clearly different from that of the young adults.

#### 3.1.2. Knee Joint Profiles

**Figure 2:**
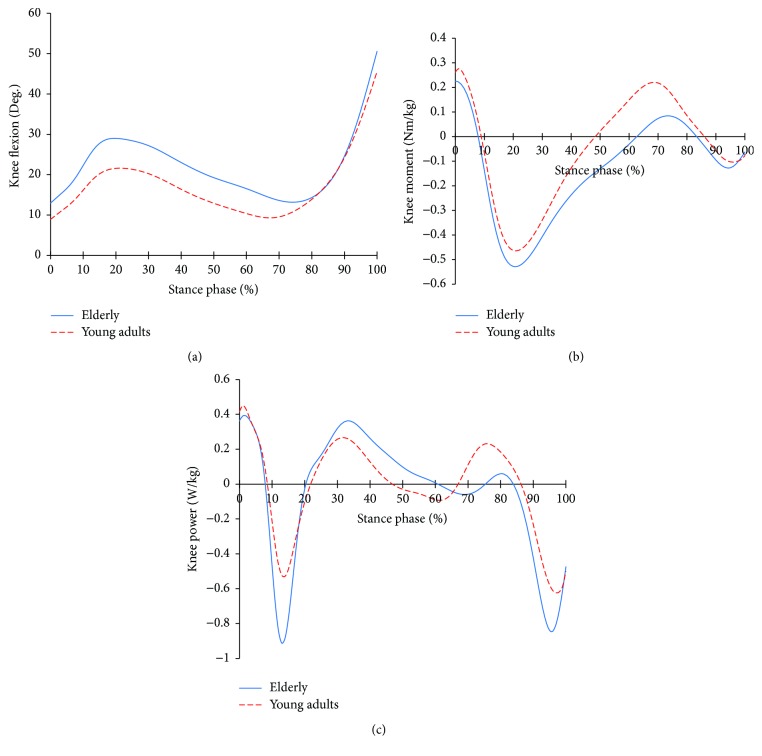
Knee joint profiles in the elderly and young adults. (a) Knee joint angle; (b) knee joint moment; (c) knee joint power.

Figure 2 shows the knee joint profiles of the two groups. In the flexion angle, both groups performed level gait with flexion. But, the elderly showed greater flexions compared to the younger group. At that moment, the moment phase change patterns were the same in the two groups. But, the timing of change was different. The elderly showed a higher extensor moment at 10~60% of stance phase and a lower flexor moment at 60~80% of stance phase compared to the young adults. In the power, likewise, the phase changes of the power of the two groups were the same. But, the timing of the change was different. And at 10~60% of stance phase, the elderly showed negative powers and positive powers which were higher than those of the young adults. Then, there were lower positive powers and higher negative powers.

#### 3.1.3. Hip Joint Profiles

**Figure 3:**
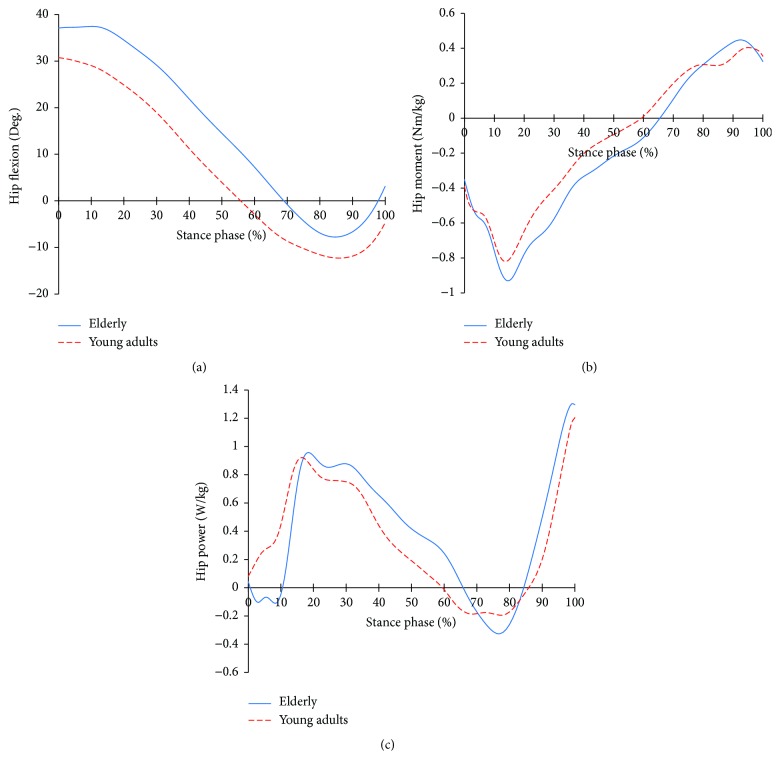
Hip joint profiles in the elderly and young adults. (a) Hip joint angle; (b) hip joint moment; (c) hip joint power.

Figure 3 shows the hip joint profiles of the two groups. In the flexion angle, the elderly showed a much higher flexion and very little extension. Moreover, the timing of changing from flexion to extension differed from that of the young adults. This means that the elder group performed level walking with their hip joint flexing; the gait period is different from that of the young adults. At that moment, the elderly showed a greater extensor moment compared to the young adults. Also, the timing that the extensor moment changed to a flexor moment was different from that of the young adults. In the power, the elder group showed energy absorption (negative power) at 0~10%. Then, they showed larger energy generation (positive power) and energy absorption compared to the young adults. As with the angles and the moments, the timing of changing phases was different from those of young adults.

#### 3.1.4. Support Moment Profiles

**Figure 4:**
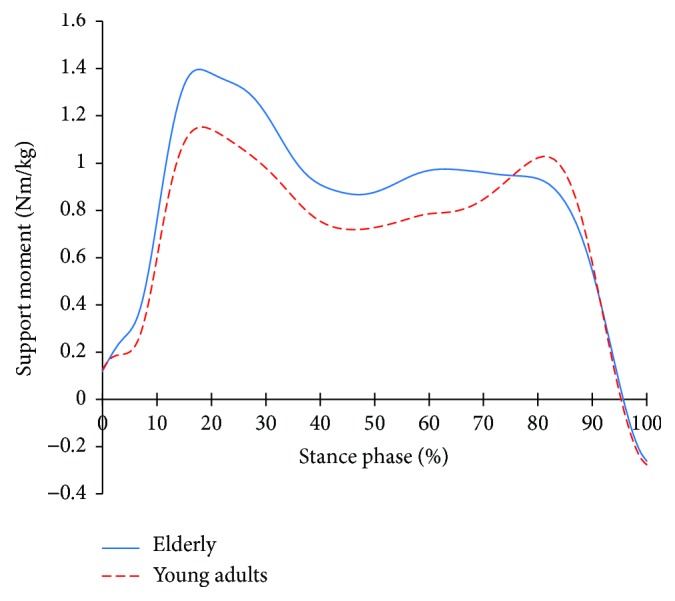
Support moment in the elderly and young adults.

**Figure 5:**
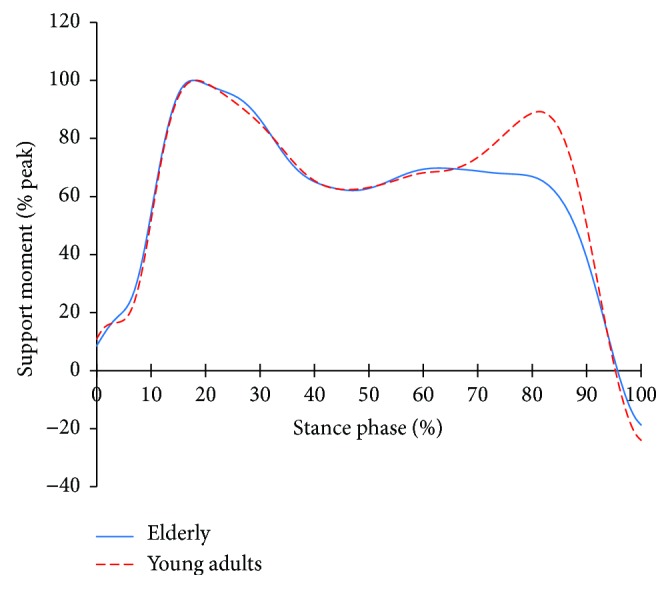
Peak normalized support moments in both groups.

Figure 4 shows the support moment profiles of the two groups, while Figure 5 shows the support moments of both groups normalized to the peak of their support moments, respectively. In Figure 4, the support moment is higher until reaching 80% of the stance phase and then lower after exceeding 80% of the stance phase than young adults. In Figure 5, the features are even more apparent. After 65% of the stance phase, the normalized support moment of the elderly is clearly lower than that of the young adults.

### 3.2. Level Gait in the Elderly during Tibialis Anterior Tendon Vibratory Stimuli

#### 3.2.1. Ankle Joint Profiles during Tibialis Anterior Tendon Vibratory Stimuli

**Figure 6:**
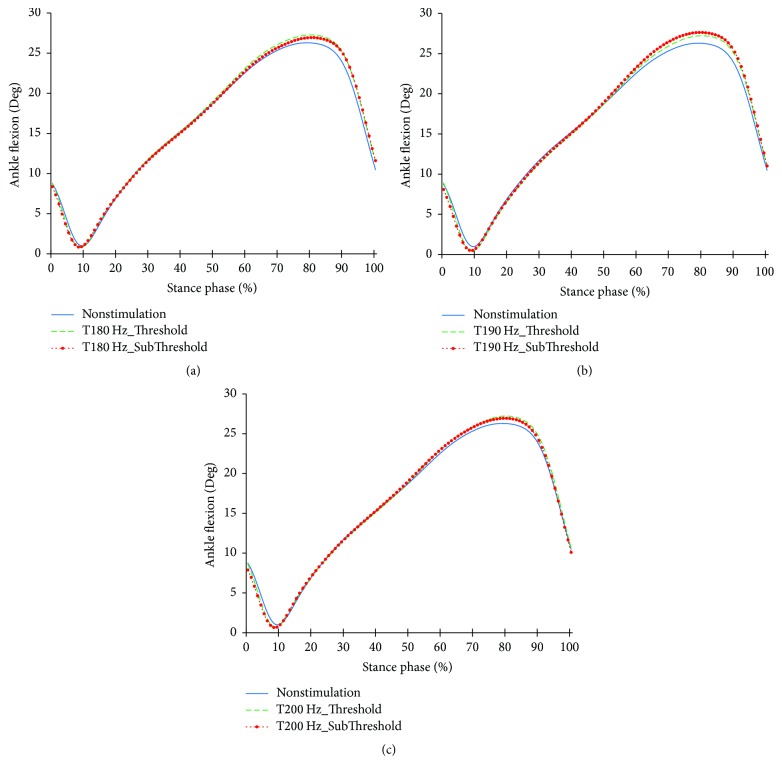
Ankle joint angle profiles in the focal vibratory stimuli conditions. (a) Ankle joint angle in the 180 Hz; (b) ankle joint angle in the 190 Hz; (c) ankle joint angle in the 200 Hz.

**Figure 7:**
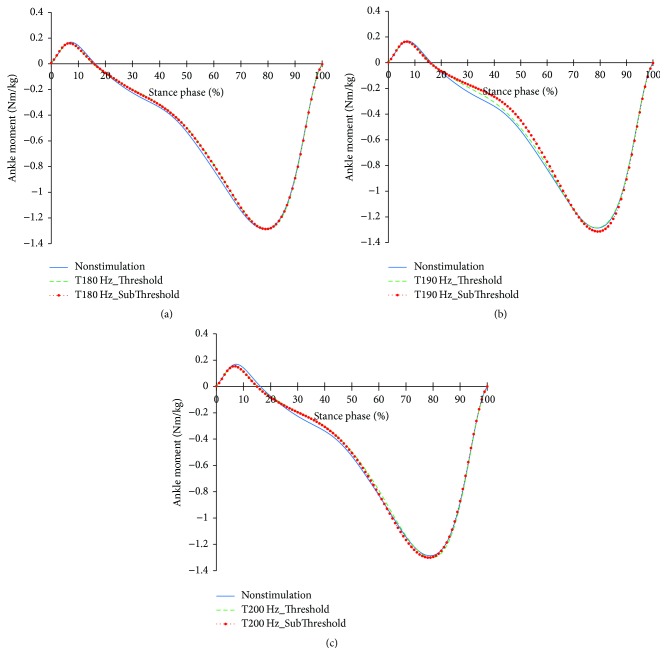
Ankle joint moment profiles in the focal vibratory stimuli conditions. (a) Ankle joint moment in the 180 Hz; (b) ankle joint moment in the 190 Hz; (c) ankle joint moment in the 200 Hz.

**Figure 8:**
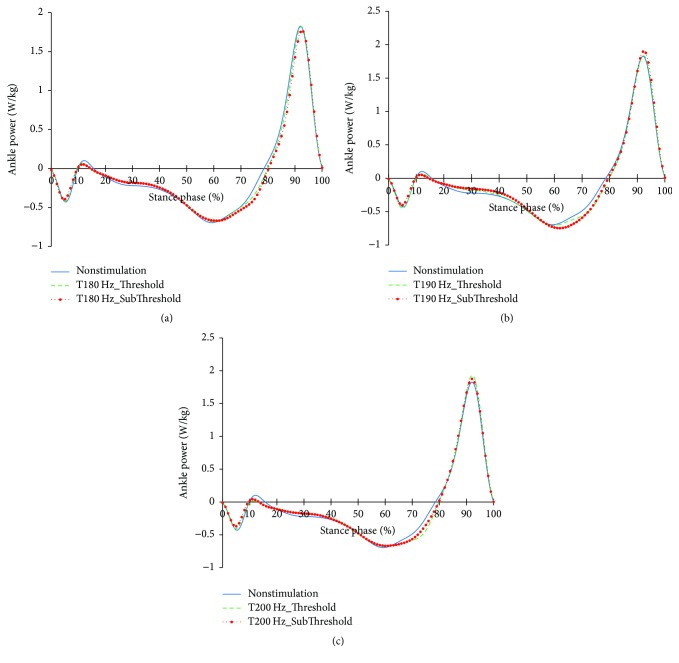
Ankle joint power profiles in the focal vibratory stimuli conditions. (a) Ankle joint power in the 180 Hz; (b) ankle joint power in the 190 Hz; (c) ankle joint power in the 200 Hz.

The profiles of the ankle joints differing due to the changes in the tibialis anterior tendon vibratory stimuli frequency and intensity are shown in Figures 6–8. As for the angles, both the reduction of dorsiflexion at 0~15% of the stance phase and the increase in dorsiflexion after 65% of the stance phase happened under focal vibratory stimuli. At that moment, the slight reduction in the dorsiflexor moment at 0~15%, the reduction in the plantar flexor moment at 20~65%, and the increase in the plantar flexor moment after 65% of the stance phase all happened under focal vibratory stimuli. In the power, the reduction in the negative power and positive power at 0~15% and the increase in the negative power at 60~80% both happened under focal vibratory stimuli conditions. Except for the 180 Hz, an increase in the positive peak power after 80% of the stance phase was observed.

#### 3.2.2. Knee Joint Profiles during Tibialis Anterior Tendon Vibratory Stimuli

**Figure 9:**
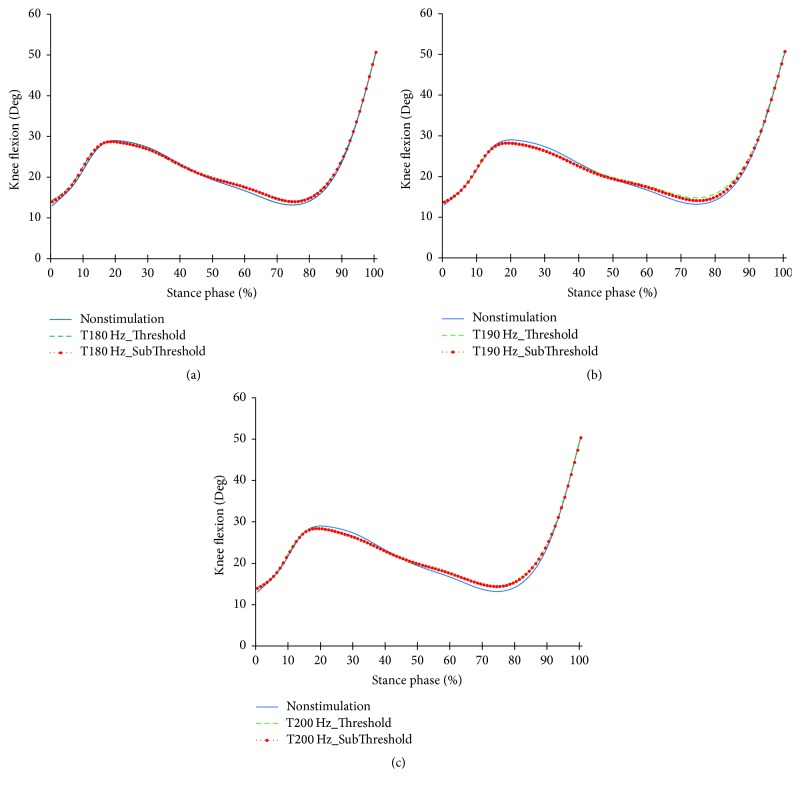
Knee joint angle profiles in the focal vibratory stimuli conditions. (a) Knee joint angle in the 180 Hz; (b) knee joint angle in the 190 Hz; (c) knee joint angle in the 200 Hz.

**Figure 10:**
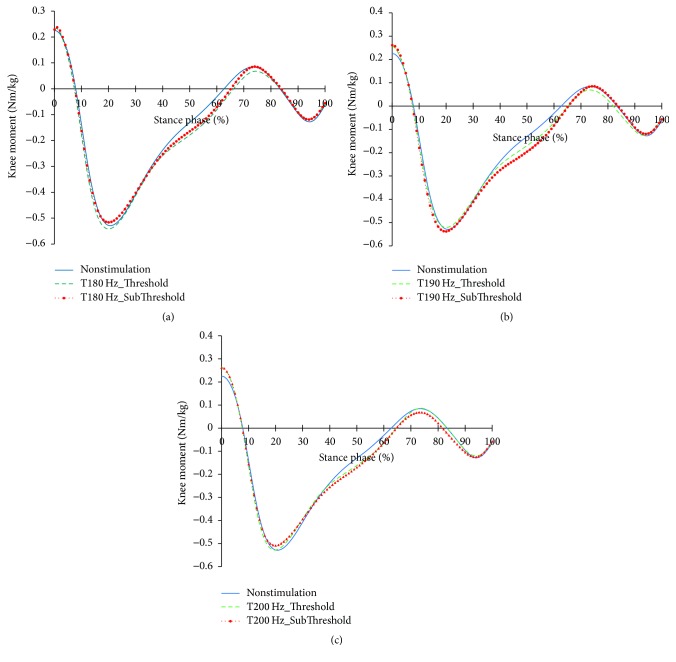
Knee joint moment profiles in the focal vibratory stimuli conditions. (a) Knee joint moment in the 180 Hz; (b) knee joint moment in the 190 Hz; (c) knee joint moment in the 200 Hz.

**Figure 11:**
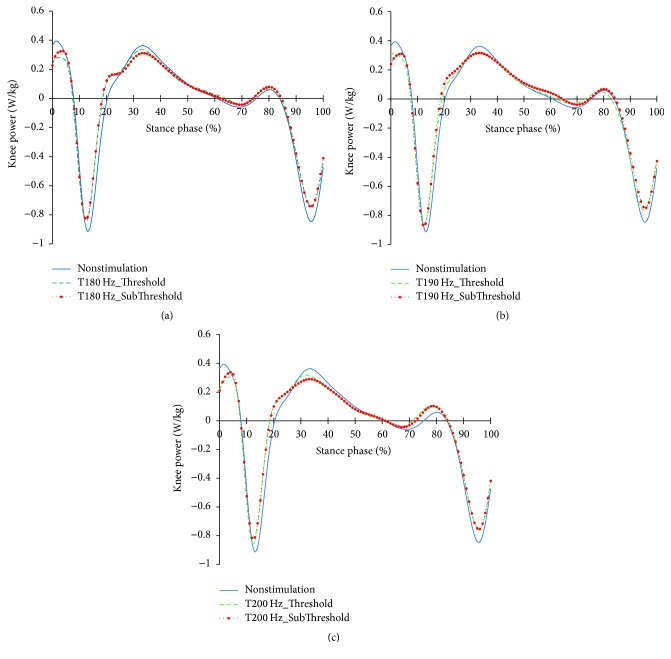
Knee joint power profiles in the focal vibratory stimuli conditions. (a) Knee joint power in the 180 Hz; (b) knee joint power in the 190 Hz; (c) knee joint power in the 200 Hz.

Figures 9–11 show the profiles of the knee joints with the frequency and intensity of tibialis anterior tendon vibratory stimuli. All focal vibratory stimuli resulted in less flexion at 15~40% and increase of flexion at 60~85%. At these moments, the flexor moment increased as the frequency became higher. And, at 35–65%, all focal vibratory stimuli resulted in an increase of the extensor moment. In the power, the positive power and the negative power reduction in the loading response phase, fast phase change timing into positive power, and the reduction of the negative power in the preswing phase happened in all focal vibratory stimuli conditions.

#### 3.2.3. Hip Joint Profiles during Tibialis Anterior Tendon Vibratory Stimuli

**Figure 12:**
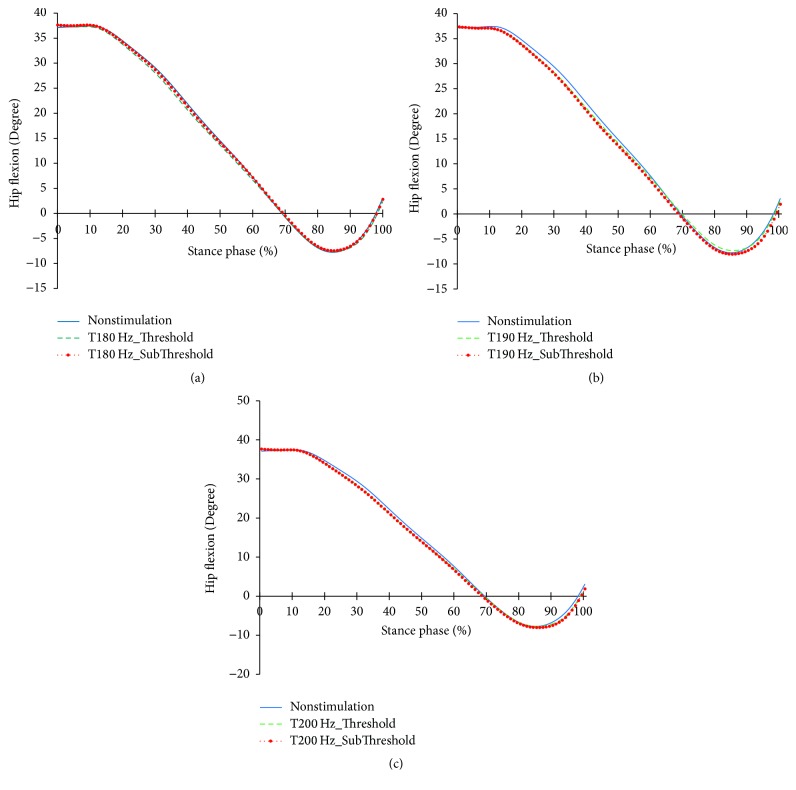
Hip joint angle profiles in the focal vibratory stimuli conditions. (a) Hip joint angle in the 180 Hz; (b) hip joint angle in the 190 Hz; (c) hip joint angle in the 200 Hz.

**Figure 13:**
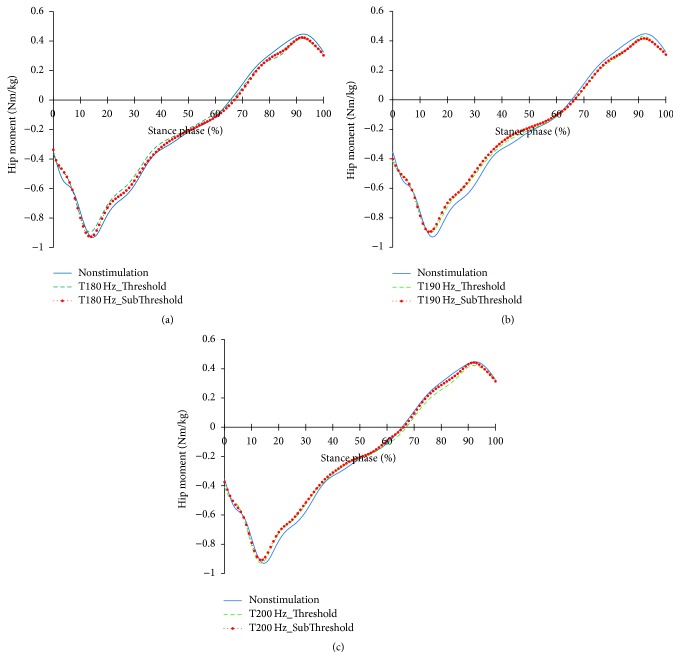
Hip joint moment profiles in the focal vibratory stimuli conditions. (a) Hip joint moment in the 180 Hz; (b) hip joint moment in the 190 Hz; (c) hip joint moment in the 200 Hz.

**Figure 14:**
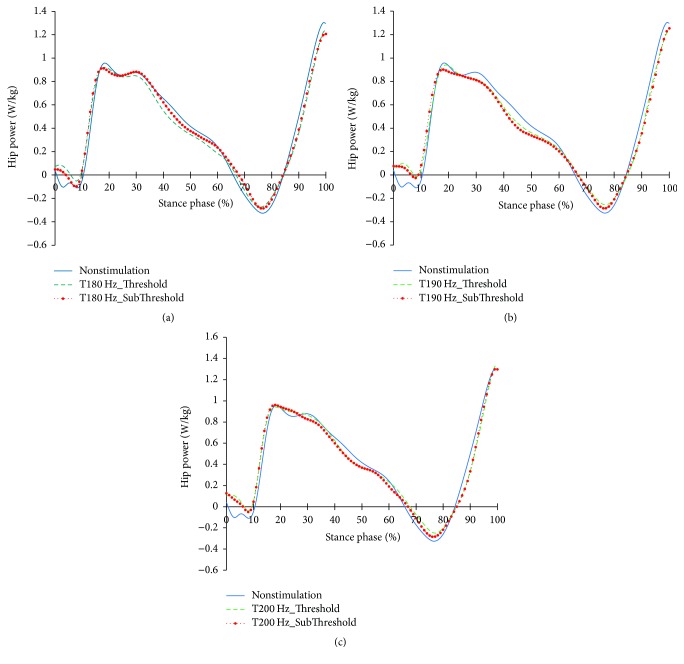
Hip joint power profiles in the focal vibratory stimuli conditions. (a) Hip joint power in the 180 Hz; (b) hip joint power in the 190 Hz; (c) hip joint power in the 200 Hz.

The profiles of the hip joints differing due to the changes in the tibialis anterior tendon vibratory stimuli frequency and strength are shown in Figures 12–14. Except for 180 Hz, the flexions of the hip joints at 15~65% (from mid-stance phase to terminal stance phase) at 190 Hz and 200 Hz both decreased. And, the extension of the hip joint at the end of the preswing phase slightly increased. In that moment, all vibratory stimuli resulted in a reduction of the extensor moment at 15–50% and a reduction of the flexor moment after 65%. In the power, positive power was observed at the loading response phase, unlike the case of nonstimulation condition. After this, all focal vibratory stimuli conditions resulted in a reduction of the positive power and the negative power from the mid-stance (MSt) to terminal stance (TSt) and a reduction in the positive power at the preswing phase (PSw).

#### 3.2.4. Support Moment Profiles during Tibialis Anterior Tendon Vibratory Stimuli

**Figure 15:**
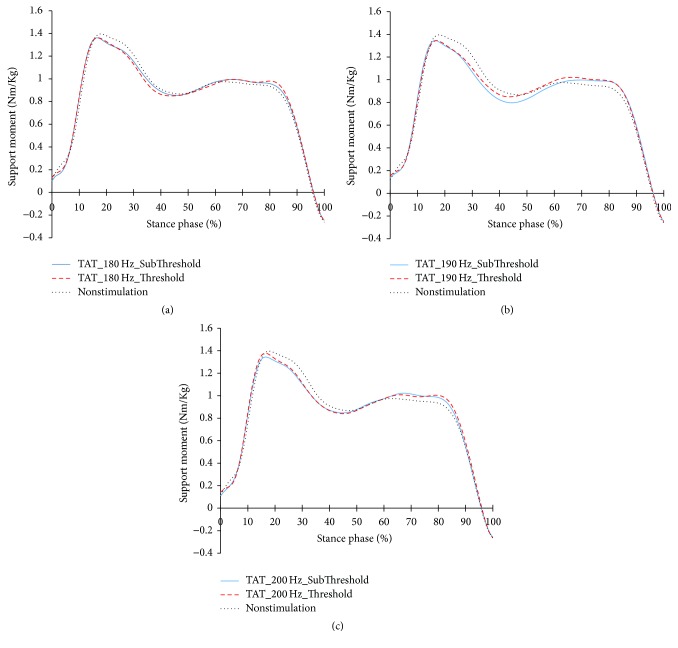
Support moments of the elderly with focal vibratory stimuli conditions. (a) Support moments of the elderly with 180 Hz focal vibratory stimuli; (b) support moment of the elderly with 190 Hz focal vibratory stimuli; (c) support moment of the elderly with 200 Hz focal vibratory stimuli.

The variations in the support moments of the elderly according to focal vibratory stimuli are shown in Figure 15. At the early stance, support moments in all focal vibratory stimuli conditions showed that they were lower than those in nonstimulation conditions. And at the late stance, the support moment is slightly higher than that of the nonstimulation condition.

### 3.3. The Vibratory Perception Threshold of the Elderly in the Tibialis Anterior Tendon

**Figure 16:**
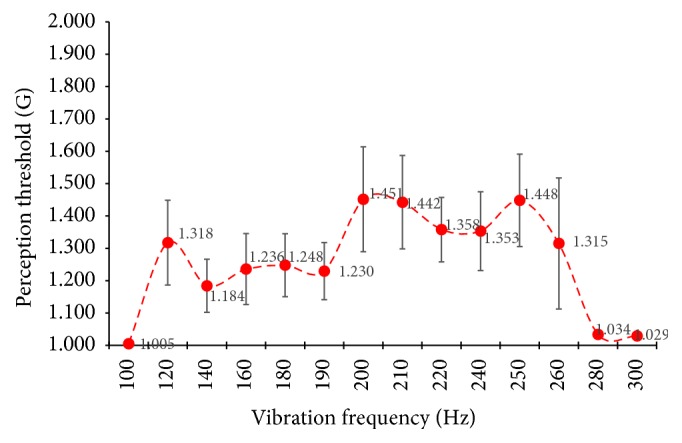
Perception threshold of the elderly with tibialis anterior tendon vibratory stimuli.

The vibratory perception thresholds were measured according to frequency in the range from 100 Hz to 300 Hz and the results are shown in Figure 16. The most sensitive vibration frequency is 190 Hz, and the vibration threshold rapidly increased at 200 Hz.

The statistical differences of the thresholds measured in the range from 180 Hz to 220 Hz are shown in Table 1. The 180 Hz has a statistical difference of 200 Hz or more frequency except for 190 Hz. And 190 Hz is the same. The 200 Hz is statistically different from all frequencies except for 220 Hz.

### 3.4. Variation of Kinematic and Kinetic Parameters When the Focal Vibratory Stimuli Applied

The differences in the joint angle profiles of the elderly and young adults according to the vibration frequency were shown in Tables 2 and 3.  Table 2 shows the mean difference in threshold intensity. The decrease in the mean difference means that the profiles of the elderly are similar to the young adults', and the results are shown in the stance and substance phases. At stance phase, the mean difference of the ankle angle was 4.31 degrees and the mean differences in the all vibration conditions were higher than that (*p* < 0.05). The same was true for the knee joints, but it was reduced for the hip joints.

For more details, in the loading response (LR), the mean differences in the ankles were reduced, whilst in the knees, they were increased. In the mid-stance (MSt), the mean differences were reduced in all joints. In the terminal-stance (TSt), the mean differences in the ankle joints and the knee joints increased, whilst in the hip joints, they were reduced. These results are almost the same even under subthreshold conditions as shown in Table 3.

The means of the support moment and joint power, according to the frequency, were shown in Tables 4 and 5. The support moment and joint power are kinetic parameters for causing movement. Therefore, to consider the functional task of the gait, the mean during single limb support (SS) and double limb support (DS) was analyzed. In particular, power is calculated by taking the absolute value [13].

In Table 4, the elderly's support moment with NS during DS was lower than that of the young adults, whilst that during SS was higher. When the focal vibratory stimuli of the threshold intensity was applied (Table 4), the support moments across all vibratory stimuli conditions increased more than those of the NS conditions of the elderly. On the other hand, the support moments during SS decreased. In the power of the ankle joints, the power of the NS condition of the elderly during DS and SS was smaller than that of the young adults. When the focal vibratory stimuli of the threshold intensity was applied (Table 4), the power during DS except 200 Hz decreased than that of the NS of the elderly, whilst during SS it increased except 180 Hz. In the power of the knee joints, the power of the elderly with NS during DS and SS was greater compared to the young adults. When the focal vibratory stimuli of the threshold intensity was applied (Table 4), the powers across all focal vibratory stimuli conditions decreased more than those of the elderly with NS. In the power of the hip joints, the power of the elderly with NS during DS and SS was greater compared to young adults. When the focal vibratory stimuli of the threshold intensity was applied (Table 4), the powers across all focal vibratory stimuli conditions decreased more than those of the elderly with NS. These results are almost the same even under subthreshold conditions as shown in Table 5.

## 4. Discussion

### 4.1. Level Gait in the Elderly

During the loading response, the dorsiflexion of the ankle joints is reduced in both groups, resulting in the foot landing on the ground. Here, the ankle joints of the elderly showed more dorsiflexion (Figure 1(a)). This is a factor weighting forward rotation of the shank, further accelerating the passive flexion of the knee joints. After the loading response, both groups showed a development of dorsiflexion, accompanied by the development of the plantar flexor moment to control the dorsiflexion and support the body. From 30%, the plantar flexor moment of the elderly continued to develop while being smaller than that of the young adults (Figure 1(b)). However, the power was similar to that of the young adults (Figure 1(c)). It seems to be a gait strategy of the elderly, which is to secure stability as, during the single limb support phase, the center of mass (COM) is lowered by increasing dorsiflexion. After this, the young adults saw a decrease in the negative power at 70% and it developed to a positive power, starting the plantar flexion of the ankle joint. However, in the elderly, the phase change of the power started at 60% (Figure 1(c)), even though the dorsiflexion was still in progress at the ankle (Figure 1(a)). This may indicate a strategy to control the accelerated dorsiflexion through a faster plantar flexion. However, this may not control sufficiently shank rotating forward caused by dorsiflexion. Then, at 80–100% of stance phase, the positive power of the elderly was lower than that of the young adults and this is consistent with previously studies [4, 14, 15]. This positive power is generated by the plantar flexor to push the body forward. The smaller positive power of the elderly is a gait strategy [4] to reduce instability in posture that could be caused by the heels elevated by plantar flexors. But, this could lead to potential impairment by decreasing muscle capacity with aging in terms of the mobile function and trunk stability [16].

Both groups showed a development of the flexion in the knee joint during the loading response (Figure 2(a)). This is the result of the action of the flexors' activities to absorb the impact force at the initial contact and the shank rotating forward over the foot. In order to control this, both groups showed an increase in the eccentric contraction of the extensors, where its magnitude was more in the elderly (Figure 2(c)). The reason for this appears to be that while the young adults would break the proceeding of the shank by a plantar flexion during the loading response, the brake of the shank is weaker with the elderly, due to the larger dorsiflexion. This seems to be a gait strategy to secure stability. However, due to the physiological weakening with aging, it can be a great burden on lengthening extensor. And there is a potential risk of a large damage when extensor suddenly extended or when failure to control length occurs. After the loading response, the extensor is still needed to achieve upright alignment and accelerate the thigh forward (Figure 2(b)). While the flexion decreased prior to the preswing phase in the elderly, the flexion was larger than that of the young adults. To support this, a larger and longer extensor moment and power compared to the young adults are required for the elderly during the single limb support phase and then smaller flexor moment occurred (Figure 2(b)). With this, it is possible to ensure stability during the single limb support phases. However, due to the reduced flexor activities, the lifting of the heel is limited, which may affect the reduced function of forward propulsion. Then, with the elevation of the heel due to the continued development of dorsiflexion and plantar flexion, the flexion of the knee joint is accelerated. To control this, the eccentric contraction of the extensor is increased (Figure 2(c)). Due to dorsiflexion increasing the knee flexion in the already large flexion state of the knee joint, the elderly had a higher negative power than that of the young adults. This can be a reason to increase the strain on the extensor.

At the hip joints, the young adult group sees a decrease in flexion, enters into an extension, and then performs a flexion again. On the other hand, the elder group maintains the flexion at the early stance. Then, while the flexion is reduced, the flexion of the elderly is larger and lasts longer compared to the younger group. Then, it is reversed to a flexion after an extension that is smaller than that of the younger group (Figure 3(a)). Hip joint power is clearly different compared to the younger group during the loading response phase (Figure 3(c)). It controls the flexion of the hip joints caused by large dorsiflexion at the ankle joint and the subsequent knee joint flexion; it helps the control of the flexion at the knee joint, as well (Figure 3(c)). However, the eccentric contraction at the knee and hip joints would increase the strain on the weakened muscles with aging. After the loading response phase, the extensor of the hip joints concentrically contracts to extend thigh. And the demands of joint moment and the power to extend the thigh are higher compared to the young adults (Figures 3(b) and 3(c)). At the late stance, the extension of the elderly is smaller compared to the young adults. This would be the result of the characteristics of the elderly, which are the increased forward inclination of the pelvis [15], contracture of the flexors [16], and, as a result, more eccentric contraction of the flexors. That is, it is a mechanism to stabilize through the flexion during the single limb support phase. As a result, while the young adult is in an extension at the toe-off (at 100% of the stance phase), the elderly are in flexion.

Compared to young adults, the gait characteristics of the elderly are that they walk as the segments are in flexion. For this, the extensors of each segment work harder. This is the gait strategy of the elderly to support their bodies bodies to prevent the collapse due to the flexion of the segments and to secure stability. Figure 4 shows the result of the support moment [17] which can depict the function of support in a comprehensive manner for the extensors of each segment.

In Figure 4, the support moment is higher than that of the young adults in the early stance. This is maintained until 85% of the stance phase, after which it reduces. That is, the elderly put more emphasis on stability through the support over the entire stance phase. However, due to aging, the elderly's physiological and neurological weakening occurs and it reduces muscle capacity, so higher support moment can be a serious strain. After 85%, the plantar flexor starts its work to create a forward propulsion. For the elderly, the activities are reduced to secure more stability [4]. As a result, the support moment after 85% may have decreased. The characteristics are more obvious; they are, respectively, normalized to the peak of the support moment of each group (Figure 5). From 65%, a rapidly peak uphill like a young adult does not appear to the elderly. This means that, at the terminal stance phase, the action of the plantar flexor becomes the priority to give support, rather than push forward. While this will limit the advancement of the lower limbs and the forward propulsion, it would be more advantageous in stabilization.

### 4.2. Changes in the Elderly Gait during Tibialis Anterior Tendon Vibratory Stimuli

When the vibratory stimuli were applied, the dorsiflexion decreased in all frequencies during the loading response (Figure 6). The reduction of dorsiflexion was a result of the decrease in the dorsiflexor moment and power (Figures 7 and 8). As the foot will be more inclined to plantar flexion compared to the nonstimulation condition, it would control the forward rotation of the shank. The dorsiflexion, which is reduced by the vibratory stimuli, would affect the flexion of the knees and hip joints caused by the excessive dorsiflexion and the eccentric contraction of the extensors to control them. After the reduction of the dorsiflexion of the ankle joint, the flexion in the knee joints decreased, and the positive and the negative powers both decreased (Figures 9 and 11). The flexion at the hip joints decreased (Figure 12). The positive power especially was generated as the negative power decreased (Figure 14). As a result, the flexion in lower limbs was reduced in general, so that the body weight would be supported in an extension state. The concentric contraction of the hip joint extensors especially relieved the burden of the eccentric, and in combination with the reduced dorsiflexion, the shock absorbing burden of the knee extensor was reduced. That is, at the early stance, the vibratory stimuli induced the elderly gait pattern in a direction to relieve the shock absorption and the body support that had been highly burdened with a large flexion of the lower extremity segment. Such a change in the elderly gait pattern was more profound at 190 Hz and 200 Hz.

After the loading response, which was followed by dorsiflexion, the hip joints' flexion kept decreasing to the direction of extension. Here, the timing of power generation for extension came earlier and smaller (Figure 14). At the knee joints, too, the flexion to the extension direction was reduced, while the power to create extension was generated sooner and smaller (Figure 11). This may be because, as the dorsiflexion was reduced, the overall flexion in the lower extremity segments was reduced, too.

Because the flexion was reduced, the beginning of the extension would be accelerated. It would also require less muscle work requirements to counteract the smaller flexion. In the end, vibratory stimuli would contribute to achieve upright alignment sooner during the single limb support phase, along with the state of extension facilitated during the loading response phase.

As the gait with vibratory stimulus progressed, the dorsiflexion increased more than that of nonstimulation since the 55% of the stance phase (Figure 6). To control this dorsiflexion, negative power of the plantar flexor lasted longer than nonstimulation condition (Figure 8). As the dorsiflexion increased, the flexion of the knee increased, resulting in an increase in extensor moment and positive power less than nonstimulation was equal to nonstimulation (Figure 11). Soon, the negative power to control the hip extension decreased (Figure 14). An increase in the dorsiflexion of the ankle joint and flexion of the knee joint will lower the center of mass, thereby ensuring stability and achieving careful forward walking. It will also improve insufficient body support during single limb support phase by rapid negative power reduction in nonstimulation condition.

After 85%, the dorsiflexion decreased, which increased knee flexion. And hip flexion reversed to extension. In focal vibratory stimuli conditions, the dorsiflexion declined to nonstimulation levels, and this tendency became more pronounced as the frequency increased. The plantar flexor moment is similar to nonstimulation, while positive power increased. This tendency became more pronounced as the frequency increased, too (Figure 8). The hip flexor moment was slightly lower than that of the nonstimulation condition (Figure 13), and positive power also decreased (Figure 14). This contributed to a reduction in the demands of the knee extensor to prevent knee collapse before toe-off. The increased activity of plantar flexor pushed the tibia further backward, and the reduced activity of the hip flexor pulls the thigh less forward. This activity caused the knee to extend. In addition, the flexion of the knee, due to the focal vibratory stimuli in the terminal stance phase, was less extensible rather than in the nonstimulation condition. In this state, the reduction of the dorsiflexion causes the flexion of the knee to reach a level of nonstimulation's flexion. This means that the reversal from extension to flexion was less than in nonstimulation, and the result will also contribute to a reduction in the negative power of the knee extensor.

When the focal vibratory stimuli were applied, the gait characteristics of the elderly were as follows: reduction of the body support at the early stance as a result of the decrease in flexion of the knee and hip by reduced dorsiflexion. At the late stance, there was an increase of the moment for single support because of increased dorsiflexion and the flexion of the knee joint. There was also a reduction in eccentric contractions of the knee extensors because of activities of the plantar flexor and the flexor of the hip. That is, the focal vibratory stimuli will change the gait of the elderly and affect the overall function of the lower extremity muscles during gait.

The changes in the function of the extensor muscles of each segment due to the focal vibratory stimuli are shown in Figure 15. Support moments decreased in the early stance and increased in the late stance in all focal vibratory stimuli conditions. In the elderly whose physiological function is weakened by aging, the reduction of support moment at the early stance means that the burden of the body support of the extensor muscles is relaxed. And the increase of the support moment at the late stance means the increase of stability during the single support phase.

### 4.3. Improvement Effects of the Focal Vibratory Stimuli

To examine improvement effects of the focal vibratory stimuli, the variations of the support moment and joint power and the similarity to angle profiles of the young adults were analyzed.

During the entire stance phase, only the hip joint angles were found to be similar to that of young adults'. However, since the movement of each segment of the lower extremity progresses sequentially and organically in the time domain, it is important to investigate it by dividing it into subperiods of the gait.

When the focal vibratory stimuli of the threshold intensity were applied in the LR, the difference in the ankle angle profiles of the young adults was decreased than that of the elderly with NS being as shown in Table 2 (*p* < 0.05). This means that the dorsiflexion in the LR is similar to the young adults, and it results from more plantar flexion by the focal vibratory stimuli. After LR, the profile difference increased (*p* < 0.05), which is the same as the result of Figure 6. This is an effect of the focal vibratory stimuli and it will contribute to the reduction of COM height as described in Section 4.2. In the knee joint, the difference in the angle profiles during LR was slightly increased. This means increased knee flexion and it is associated with shock absorption. The knee flexion and flexor moment in the LR are intended to absorb the impact force [13]. Therefore, this result reveals that the focal vibratory stimuli positively contributed to the shock absorption function of the knee joint muscles.

In the MSt, the profile differences of the knee angle were reduced, which is due to the reduction of the dorsiflexion in the LR as described in Section 4.2. The difference in the profiles after MSt increased. This is the result of the dorsiflexion, which is increased by the vibratory stimuli and the increase of the stability assurance strategy. In the hip joints, the difference in profiles was reduced in all subperiods of the gait as shown in Table 2 (*p* < 0.05). This means that the focal vibratory stimuli leads the profile of the elder's hip joint angle toward that of the young adults. This means that, like young adults, the hips joints walk in a more extended state, which will certainly contribute to a reduction in power to support the body.

From these results, it can be seen that the joint angles, which are kinematic variables of the gait, are induced to the profiles of the young adults by the focal vibratory stimuli in the subperiods as the gait progresses. These results are also found in the subthreshold intensity as shown in Table 3.

In the support moments of Table 4, the support moments of the elderly with NS during DS are smaller than those of the young adults (*p* < 0.05). This will be due to the low support moments at the PSw (Figure 15). This is a strategy to secure a more stable gait, but it is a negative factor for forward propulsion. When the focal vibratory stimuli were applied, all the support moments increased (*p* < 0.05). This will contribute more to stability than forward propulsion. In order to facilitate forward propulsion, COM should be accelerated through extension of the lower extremity. However, the ankle and knee flexions increased in the late stance (Figures 6 and 9). Therefore, the focal vibratory stimuli will further promote the stability of the gait.

The support moments of the elderly with NS during SS were higher than the young adults' (*p* < 0.05). This means that the activity of the extensors of the lower extremity is greater than that of the young adults during single limb stance, as shown in Figure 5. This means that, in order to perform functional tasks during the SS, the recruitment of the extensors of the lower extremity is much higher than the young adults', and the energy demands, therefore, will also be high. This is evident in the power of the knees and hips even during DS (*p* < 0.05). Considering the neurophysiological weakening caused by aging of the elderly, this will be a great burden. However, when the focal vibratory stimuli were applied, both the support moments and the power of the knee and hip joints decreased during SS (*p* < 0.05), and the same results were obtained during DS. This means that the activity of the extensor for the gait and the corresponding energy demand decreased. As a result, the burden of proceeding the gait is alleviated. In addition, considering that the joint angles gradually became similar to the young adults' during the subperiods of the gait (Table 2), it can be inferred that the efficiency of gait progression in the neurophysiologic aspect is increased.

### 4.4. The Elderly Gait Depending on Vibration Intensity and Frequency

Vibratory stimulation can generate evoked cortical potentials in sensory and motor cortical areas [10]. When direct high frequency vibration is applied, cortical areas receive and process proprioception, which generates evoked cortical potentials [11, 18]. Forner-Cordero et al. [10] examined changes in corticomotor excitability. They are applied to dominant distal wrist flexor tendon and then the amplitude of Motor evoked potential (MEP) is measured. They reported that MEP amplitude for dominant flexor carpi radialis increased significantly. Therefore, the change in the elderly gait pattern due to the focal vibratory stimuli may be the result of the focal vibration stimulation affecting the central nervous system.

In this study, the elderly gait was changed according to the frequency and stimulus intensity of the applied vibration. In stimulation intensity condition, when the vibration of a 180 Hz threshold intensity was applied, the kinematic and kinetic variables of the elderly gait changed. Even at subthreshold intensity conditions of 180 Hz, the variables changed, and the change pattern was almost similar to the change pattern at the threshold intensity condition. A similar pattern of changes in threshold and subthreshold intensities at 180 Hz frequency also occurred at the 190 Hz and 200 Hz vibration stimuli. In our previous study [19], we investigated the changes in somatosensory evoked potentials (SEPs) according to the stimulus intensity at each vibration frequency. As a result, when the stimulus intensity was 80% of the threshold intensity (increasing 5%), the SEPs of the vibratory stimuli were significantly different from the SEPs of the nonstimulation. There was no significant difference between the stimulus intensity of 80% or more. Therefore, the reason why the change in the subthreshold pattern and the change in the threshold pattern are similar is due to the potential in the somatosensory area.

In stimulation frequency condition, the change of the support moment differs depending on the vibration frequency. This implies that the function of the extensor muscles of the supporting legs changes according to the vibration frequency. This may be because the excitability of the central nervous system is dependent on the frequency of vibration. Steyvers et al. [20] investigated corticospinal excitability according to the frequency of muscle tendon vibration. They measured the MEP according to the frequency of vibration using transcranial magnetic stimulation (TMS) and reported that muscle tendon vibration exerts a frequency-dependent effect on corticospinal excitability.

We investigated the sensory threshold of vibration according to the vibration frequency of the elderly (a total of 11 elderly), illustrated in Figure 16. The vibration threshold values from 100 Hz to 300 Hz were measured. As a result, only one can feel the vibrations of 100 Hz, 280 Hz, and 300 Hz, and there are more than 7 people who cannot feel the vibration of 120 Hz~160 Hz and 240 Hz~260 Hz. So, as can be seen in Figure 16, the most sensitive vibration frequency is 190 Hz, and the vibration threshold is rapidly increased at 200 Hz.

Table 1 shows the statistical differences of the vibration threshold values from 180 Hz to 220 Hz. 180 Hz has a statistical difference of 200 Hz or more except for 190 Hz, and 190 Hz is the same. On the other hand, 200 Hz is statistically different from all frequencies except for 220 Hz. Sensory perception thresholds are related to the peripheral sensory receptors and thus the sensory area of the cerebral cortex. In other words, the fact that the vibration threshold varies according to the frequency of the applied vibration suggests that the response of the central nervous system will be different depending on the frequency of the vibration, and accordingly, the activity of the motor may be frequency-dependent.

### 4.5. Benefits and Risks of the Focal Vibratory Stimuli

In this study, we applied the focal vibratory stimuli to the tibialis anterior tendon of the elderly during gait and confirmed that the focal vibratory stimuli affect the kinematic and kinetic gait of the elderly and induce variations in the gait profiles. As a result, it was found that the focal vibratory stimuli positively contribute to the relaxation of neurophysiological demands for gait performance and the efficiency of gait progression.

The focal vibration applied in this study is at least 6 seconds, which is a very short time. Therefore, the results of this study show that the focal vibratory stimuli have acute effects. As shown in this study, many studies have revealed acute effects of vibration stimulation and are well described in the study of Luo et al. [9]. There are various vibration applying times that are not only from seconds to minutes but also over 1 hour [21]. Also, some studies have applied up to 12 weeks (3 month). These studies have shown that vibratory stimulation contributes positively to EMG, muscle force, and neuromuscular performance. Lapole and Pérot [22, 23] reported that the tendon-vibration program for two weeks increased triceps surae force production and also reduced stiffness and reflexes and said that the vibration stimulation could be beneficial to immobilized persons as hypo-activity.

There are more recent studies that summarize the effects of the focal vibratory stimuli only [24], and the vibration applying time is from 6 seconds to 12 weeks. Constatino et al. [25] applied local vibration for 4 weeks to chronic poststroke patients and found statistically significant improvement in grip muscle strength, pain, and quality of life and a decrease in spasticity and said that local muscle vibration treatment might be an additional and safe tool in the management of chronic poststroke patients, granting its high therapeutic efficiency, limited cost, and short and repeatable protocol of use. Camerota et al. [26] applied repeated focal muscle vibration (r-fMV) to patients with severe gait impairment due to multiple sclerosis for 30 minutes; this was repeated for 3 consecutive days. They measured the effect of the repeated focal muscle vibration on gait by a gait analysis that was performed before r-fMV (T0) and 1 week (T1) and 1 month (T2) after the last session of r-fMV. They reported that, after the r-fMV, the most of spatiotemporal parameters improved multiple sclerosis patients' quality of life. Also, they concluded that r-fMV improves gait function in multiple sclerosis patients.

Currently, studies using vibratory stimulation for more than one year are rare. Although the long-term effect of more than one year is difficult to clarify in this study, it is expected that positive effects will be possible through long-term application of vibration through previous studies applying vibration from a week to a month. The effect of long-term application of vibration can be estimated through functional electrical stimulation (FES). Because the external stimuli are different from each other, the stimuli delivered in the human body are the same as the electrical impulses through the nerves.

Kern et al. [27] performed home-based daily training by functional electrical stimulation (H-FES) on 25 patients suffering complete lower motor neuron paraplegia, and they investigated results before H-FES and 1 year and 2 years after it. They reported that after 1 year of H-FES, there were increases in muscle excitability and contractility and in 26% of muscle bulk and that myofiber size increased after 2 years of H-FES. Therefore, more than one year of vibratory stimulation may have similar effects to those studies' results. Furthermore, it is expected that the range of clinical applications of vibratory stimulation will be broadened through studies [28–30] that have proven to be effective in brain rehabilitation through vibration stimulation.

The various effects of the vibratory stimuli have been revealed, and potential risks must be considered in order to use them for clinical rehabilitation and therapeutic purposes. By applying vibration for a long time, the vibration perception threshold can be increased [31]. This is due to “sensory adaptation” by sustained or repetitive stimulation [31]. As the threshold increases, the vibratory stimulation initially presented can no longer activate the sensory system, so it is difficult for the effects suggested by related studies to occur. Another risk is skin keratinization at sites where vibration is repeated continuously or repeatedly. Continuous friction, pressure, and irritation accelerate skin's keratinization and, if prolonged, cause callus [32]. Because of the callus, the threshold of sensation will increase; eventually, it will be required to stop the application of vibrations or change the site stimulated. Finally, there is a potential risk to the perception threshold intensity. Sensory stimulation that is suddenly felt on the distal side during gait may cause confusion in the progress of the gait or cause sudden changes (i.e., unwanted movements). In addition, suprathreshold above the threshold may result in discomfort or loss of balance [33, 34]. In this study, subthreshold stimuli showed similar effects to threshold stimuli (Tables 2–5). Therefore, it is appropriate to apply subthreshold intensity vibration to movements like gait.

## 5. Conclusion

The purpose of this study was to identify the gait profiles of the elderly with aging, examine variations of gait parameters of the elderly by the focal tendon vibratory stimulation, and determine the effects of the focal tendon vibratory stimulation on the elderly gait. And the results were as follows:

  The elderly walk as the segments are in flexion. Because of this, the extensors of the lower limbs work harder specifically in the early stance.
  When the focal vibratory stimuli were applied, the kinematic and kinetic parameters were affected, resulting in relieving the neurophysiological demands to conduct gait.
  The response of the central nervous system to vibration is dependent on the frequency. Accordingly, the activity of the motor may be dependent on vibration stimulation characteristics.

## Figures and Tables

**Table 1:** *p*-value of the perception threshold with focal vibratory stimuli.

*p*-value	180 Hz	190 Hz	200 Hz	210 Hz	220 Hz
180 Hz	—	0.075	0.001	0.001	0.008
190 Hz	0.075	—	0.002	0.004	0.002
200 Hz	0.001	0.002	—	0.009	0.079
210 Hz	0.001	0.004	0.009	—	0.362
220 Hz	0.008	0.002	0.079	0.362	—

**Table 2:** Angle profiles' differences between the young adults and the elderly with NS and between the young adults and the elderly with all vibratory stimuli conditions of the threshold intensity (mean ± SD).

	Stance phase	LR	MSt	TSt	PSw
*Ankle joint*					
Elderly with NS	4.31 ± 1.32	0.84 ± 0.21	3.20 ± 0.16	4.05 ± 0.12	8.60 ± 1.38
with 180 Hz	*4.73* ± *1.54*^*∗*^	0.46 ± 0.27^**∗**^	*3.28* ± *0.16*^*∗*^	*4.63* ± *0.23*^*∗*^	*9.79* ± *1.40*^*∗*^
with 190 Hz	*4.48* ± *1.54*^*∗*^	0.37 ± 0.19^**∗**^	2.86 ± 0.18^**∗**^	*4.48* ± *0.26*^*∗*^	*9.59* ± *1.34*^*∗*^
with 200 Hz	*4.56* ± *1.48*^*∗*^	0.44 ± 0.26^**∗**^	3.14 ± 0.15^**∗**^	*4.49* ± *0.23*^*∗*^	*9.43* ± *1.29*^*∗*^

*Knee joint*					
Elderly with NS	4.89 ± 1.22	4.83 ± 0.35	7.00 ± 0.18	4.79 ± 0.99	1.41 ± 0.82
with 180 Hz	*5.24* ± *1.09*^*∗*^	*5.71* ± *0.32*^*∗*^	6.70 ± 0.28^**∗**^	*5.57* ± *0.90*^*∗*^	*1.84* ± *0.71*^*∗*^
with 190 Hz	*5.31* ± *0.95*^*∗*^	*5.03* ± *0.29*^*∗*^	6.41 ± 0.19^**∗**^	*5.95* ± *0.82*^*∗*^	*2.41* ± *0.67*^*∗*^
with 200 Hz	*5.22* ± *1.02*^*∗*^	*5.39* ± *0.33*^*∗*^	6.56 ± 0.28^**∗**^	*5.59* ± *0.88*^*∗*^	*2.13* ± *0.69*^*∗*^

*Hip joint*					
Elderly with NS	8.41 ± 1.05	7.27 ± 0.34	10.00 ± 0.28	8.77 ± 1.02	5.63 ± 0.57
with 180 Hz	7.91 ± 0.87^**∗**^	*7.32* ± *0.25*	9.07 ± 0.15^**∗**^	8.38 ± 0.84^**∗**^	5.35 ± 0.41^**∗**^
with 190 Hz	8.04 ± 0.84^**∗**^	7.12 ± 0.23	9.07 ± 0.22^**∗**^	8.68 ± 0.80	*5.64* ± *0.40*
with 200 Hz	7.93 ± 0.92^**∗**^	*7.46* ± *0.24*^*∗*^	9.21 ± 0.16^**∗**^	8.39 ± 0.87^**∗**^	5.15 ± 0.38^**∗**^

SD: standard deviation; NS: nonstimulated; LR: loading response; MSt: midstance; TSt: terminal-stance; PSw: preswing; the italic text indicates the increase in the profiles' differences; the bold text indicates the decrease in the profiles' differences. ^*∗*^Statistical differences (*p* < 0.05).

**Table 3:** Angle profiles' differences between the young adults and the elderly with NS and between the young adults and the elderly with all vibratory stimuli conditions of the subthreshold intensity (mean ± SD).

	Stance phase	LR	MSt	TSt	PSw
*Ankle joint*					
Elderly with NS	4.31 ± 1.32	0.84 ± 0.21	3.20 ± 0.16	4.05 ± 0.12	8.60 ± 1.38
with 180 Hz	*4.59* ± *1.55*^*∗*^	0.55 ± 0.28^**∗**^	3.21 ± 0.12	4.03 ± 0.20^*∗*^	*9.74* ± *1.50*^*∗*^
with 190 Hz	*4.77* ± *1.56*^*∗*^	0.57 ± 0.18^**∗**^	3.04 ± 0.17^**∗**^	*4.87* ± *0.31*^*∗*^	*9.92* ± *1.30*^*∗*^
with 200 Hz	*4.55* ± *1.35*^*∗*^	0.68 ± 0.20^**∗**^	*3.29* ± *1.22*^*∗*^	*4.50* ± *0.16*^*∗*^	*8.95* ± *1.22*^*∗*^

*Knee joint*					
Elderly with NS	4.89 ± 1.22	4.83 ± 0.35	7.00 ± 0.18	4.79 ± 0.99	1.41 ± 0.82
with 180 Hz	*5.26* ± *1.13*^*∗*^	*5.53* ± *0.39*^*∗*^	6.85 ± 0.25^**∗**^	*5.55* ± *0.97*^*∗*^	*1.82* ± *0.76*^*∗*^
with 190 Hz	*5.02* ± *1.03*	*5.08* ± *0.35*^*∗*^	6.34 ± 0.25^**∗**^	*5.46* ± *0.90*^*∗*^	*1.90* ± *0.73*^*∗*^
with 200 Hz	*5.23* ± *1.03*^*∗*^	*5.28* ± *0.34*^*∗*^	6.54 ± 0.24^**∗**^	*5.75* ± *0.88*^*∗*^	*1.97* ± *0.60*^*∗*^

*Hip joint*					
Elderly with NS	8.41 ± 1.05	7.27 ± 0.34	10.00 ± 0.28	8.77 ± 1.02	5.63 ± 0.57
with 180 Hz	8.34 ± 0.93^**∗**^	*7.58* ± *0.30*^*∗*^	9.65 ± 0.20^**∗**^	*8.79* ± *0.90*	5.70 ± 0.45
with 190 Hz	7.59 ± 0.89^**∗**^	7.18 ± 0.25	8.81 ± 0.13^**∗**^	8.01 ± 0.86^**∗**^	4.92 ± 0.39^**∗**^
with 200 Hz	7.75 ± 0.96^**∗**^	*7.52* ± *0.26*^*∗*^	9.14 ± 0.17^**∗**^	8.10 ± 0.90^**∗**^	4.84 ± 0.38^**∗**^

SD: standard deviation; NS: nonstimulated; LR: loading response; MSt: midstance; TSt: Terminal-stance; PSw: preswing; the italic text indicates the increase in the profiles' differences; the bold text indicates the decrease in the profiles' differences. ^*∗*^Statistical differences (*p* < 0.05).

**Table 4:** Mean differences between the young adults and the elderly with NS and between the elderly with NS and the elderly with all vibratory stimuli conditions of the threshold intensity (Mean ± SD).

	Young adults		Elderly with NS	With 180 Hz	With 190 Hz	With 200 Hz
Support moment						
DS	0.450 ± 0.167	>	0.445 ± 0.144	*0.469* ^+^ ± *0.160*	*0.463* ^+^ ± *0.158*	*0.474* ^+^ ± *0.162*
SS	0.884 ± 0.070	<	1.045^*∗*^ ± 0.088	^**∗**^1.028^+^ ± 0.080	^**∗**^1.032 ± 0.073	^**∗**^1.035 ± 0.080
Ankle power						
DS	1.208 ± 0.524	>	0.662^*∗*^ ± 0.301	^**∗**^0.639^+^ ± 0.300	^**∗**^0.657 ± 0.303	^*∗*^ *0.690* ^+^ ± *0.321*
SS	0.378 ± 0.121	>	0.329^*∗*^ ± 0.105	^**∗**^0.322 ± 0.106	^*∗*^ *0.338* ± *0.115*	^*∗*^ *0.338* ± *0.113*
Knee power						
DS	0.302 ± 0.100	<	0.375^*∗*^ ± 0.134	0.333^+^ ± 0.119	0.351^+^ ± 0.121	0.341^+^ ± 0.120
SS	0.156 ± 0.064	<	0.193^*∗*^ ± 0.109	0.175^+^ ± 0.096	0.176^+^ ± 0.101	0.176^+^ ± 0.096
Hip power						
DS	0.368 ± 0.174	<	0.423^*∗*^ ± 0.226	0.354^+^ ± 0.208	0.360^+^ ± 0.209	0.371^+^ ± 0.222
SS	0.413 ± 0.149	<	0.505^*∗*^ ± 0.145	^**∗**^0.467^+^ ± 0.145	^**∗**^0.467^+^ ± 0.145	^**∗**^0.490^+^ ± 0.153

SD: standard deviation; NS: nonstimulated; the italic text indicates the increase in the mean; the bold text indicates the decrease in the mean; ^*∗*^Statistical differences with the young adults (*p* < 0.05). ^+^Statistical differences with the elderly with NS (*p* < 0.05).

**Table 5:** Mean differences between the young adults and the elderly with NS and between the elderly with NS and the elderly with all vibratory stimuli conditions of the subthreshold intensity (Mean ± SD).

	Young adults		Elderly with NS	With 180 Hz (Sub-thres.)	With 190 Hz (Sub-thres.)	With 200 Hz (Sub-thres.)
Support moment						
DS	0.450 ± 0.167	>	0.445 ± 0.144	*0.455* ± *0.157*	*0.470* ^+^ ±* 0.160*	*0.455* ± *0.157*
SS	0.884 ± 0.070	<	1.045^*∗*^ ± 0.088	^**∗**^1.035^+^ ± 0.078	^**∗**^1.008^+^ ± 0.081	^**∗**^1.032 ± 0.075
Ankle power						
DS	1.208 ± 0.524	>	0.662^*∗*^ ± 0.301	^**∗**^0.606^+^ ± 0.291	^*∗*^ *0.669* ± *0.317*	^*∗*^ *0.671* ± *0.317*
SS	0.378 ± 0.121	>	0.329^*∗*^ ± 0.105	^**∗**^0.328 ± 0.106	^**∗**^0.327 ± 0.115	^*∗*^ *0.330* ± *0.113*
Knee power						
DS	0.302 ± 0.100	<	0.375^*∗*^ ± 0.134	0.338^+^ ± 0.116	0.339^+^ ± 0.118	0.341^+^ ± 0.117
SS	0.156 ± 0.064	<	0.193^*∗*^ ± 0.109	0.171^+^ ± 0.093	0.183 ± 0.097	0.170^+^ ± 0.091
Hip power						
DS	0.368 ± 0.174	<	0.423^*∗*^ ± 0.226	0.362^+^ ± 0.209	0.353^+^ ± 0.209	0.372^+^ ± 0.224
SS	0.413 ± 0.149	<	0.505^*∗*^ ± 0.145	^**∗**^0.491^+^ ± 0.147	^**∗**^0.464^+^ ± 0.142	^**∗**^0.488^+^ ± 0.151

SD: standard deviation; NS: nonstimulated; the italic text indicates the increase in the mean; the bold text indicates the decrease in the mean. ^*∗*^Statistical differences with the young adults (*p* < 0.05).^+^Statistical differences with the elderly with NS (*p* < 0.05).
